# Dietary Intake and Lifestyle Habits of Children Aged 10–12 Years Enrolled in the School Lunch Program in Greece: A Cross Sectional Analysis

**DOI:** 10.3390/nu13020493

**Published:** 2021-02-03

**Authors:** Olga Malisova, Antonis Vlassopoulos, Aikaterini Kandyliari, Evaggelia Panagodimou, Maria Kapsokefalou

**Affiliations:** Department of Food Science and Human Nutrition, Agricultural University of Athens, 11855 Athens, Greece; olgamalisova@yahoo.gr (O.M.); avlassopoulos@aua.gr (A.V.); kkandyliari@aua.gr (A.K.); evagelia93@hotmail.com (E.P.)

**Keywords:** School Lunch, eating habits, sedentary lifestyle, school-aged children

## Abstract

School Lunch programs are a common strategy to address social inequalities in food access among children, especially food insecurity. The aim of this study was to evaluate the dietary intake and lifestyle habits of children aged 10–12 years enrolled in the School Lunch Program in Greece. A cross-sectional survey of fifth and sixth grade students, School Lunch recipients (n = 609) and control subjects (n = 736), collected data on sociodemographic, nutritional and lifestyle habits via self-reported questionnaires during May–October 2019. Despite enrollment in the School Lunch Program children in this group reported consuming less meals during the day (3.47 ± 1.38 vs. 3.65 ± 1.35, *p* = 0.002). No differences were seen in intakes of energy and macronutrients, however School Lunch recipients reported lower intakes of cereals/potatoes and legumes but higher fruit intake (2.32 ± 1.59 vs. 1.97 ± 1.72, *p* < 0.05). School Lunch recipients reported 42min/d and 28min/d higher screen-time during weekdays and weekends, respectively. Linear regression highlighted that dietary quality was not associated with School Lunch enrollment but rather sleep duration and screen time had a stronger influence on dietary habits. Enrollment in a School Lunch Program was linked to sustained differences in sedentary lifestyle habits but less so in dietary habits.

## 1. Introduction

A key focus of school level nutritional policies is ensuring that children at school have access to healthy, nutritious foods through the promotion of a healthy food environment [1]. To that extent, two policies are the most commonly employed, (i) the regulation of foods available for purchase in school canteens and/or (ii) the provision of school meals, either free or with a small cost [1,2]. School Lunch, the most effective of all policies, is often seen as a strong political commitment in the fight of childhood obesity and is often introduced in countries/states with an extensive portfolio of public health measures [3]. 

The effectiveness of school meals in improving dietary habits is often linked to their capacity to safeguard children against the purchase of less healthy food alternatives from vendors outside and inside the school, even in the presence of regulations around foods in the school canteen [1,4]. However, the strong argument in favor of school meals is that they can alleviate socioeconomic inequalities in food and health, as it is the children in the lower socioeconomic classes that benefit the most from being enrolled in a school meal program [5,6,7,8]. 

Over the past decade, the economic crisis has undermined children’s health, disproportionately affecting the families of children from the most vulnerable groups both at home and at school, increasing their risk of any form of malnutrition [8,9,10]. With food security on the rise, the Greek government introduced School Lunches as a state intervention in 2016 in an effort to ensure access to sufficient nutritious food for children affected by the economic crisis [11]. The National School Lunch Program was initially launched as a pilot among areas of high deprivation and was expanded in 2018 to cover 1227 primary schools around Greece (Ministerial Decision for 2018 Φ.14/ΦΜ/133730/Δ1/2018, ΦΕΚ 3508/Β/21-8-2018). The program, which operates in public schools only, offers one hot meal (lunch) daily to each student, following a biweekly meal plan designed by the Agricultural University of Athens according to the principles of the Mediterranean diet and the National Food Based Guidelines. Public schools can opt to be enrolled in the program in the beginning of each academic year based on socio-economic indicators of the vicinity such as of poverty and parental unemployment. For schools enrolled in the program, meals are provided to all children irrespectively of the household socioeconomic status, as long as their parents/guardians have given their written consent and indicative information on food allergies or intolerances of their children.

State driven nutrition interventions are a recent development in Greece, mainly after the economic crisis. School-level nutrition interventions are so far rare, as public schools do not include any structured food provision at any time during the day, unlike other European countries. Monitoring and evaluation of these new developments in nutrition policy is important, especially in countries with limited history in such initiatives. Previous data from the evaluation of the state Food Bank initiative, implemented in the framework of the Fund for European Aid to the Most Deprived (FEAD), provided with important data on the dietary habits, nutritional intake and lifestyle determinants of the most deprived in Greece and identified potential areas of improvement [12]. So far, there is a critical gap in characterizing the dietary habits and lifestyle determinants of children enrolled in the School Lunch Program to guide its design, future implementation and even the need for a structured approach in food provision as part of the school environment. 

The present study aimed to better characterize the dietary habits, nutritional intake and lifestyle determinants of children enrolled in the School Lunch Program in Greece as compared to an age matched control from the same vicinity.

## 2. Materials and Methods 

A cross sectional study was carried out in 2019 during the May–June and September–October time-periods. The research methodology followed, was approved by the Agricultural University of Athens Research Committee on Research Ethics and Conduct (28, 10-05-19) and the Hellenic Ministry of Education, Department of Primary Education (Φ.14/ΦΜ/46270/50452/Δ1, 02-04-19) as required by the Greek law for any study conducted in the school environment, during formal school hours. 

### 2.1. Participants and Study Design

The study design included the following protocol. A list of schools provided by the Ministry of Education was used to identify schools across Greece that participated in the School Lunch Program as of September 2019. Schools were selected based on the geographical distribution of the Greek population. An important inclusion criterion was the coexistence of schools participating in the School Lunch Program and control schools in the same vicinity. The Ministry of Education was provided with clearance to enter the schools which was approved by the principal of each school individually. Participant recruitment was carried out in two stages. After receiving written clearance from the school principal, researchers organized a screening visit in the school. During the screening visit, researchers provided the students with the parent and student questionnaires gave instructions for completion and explained the study process to the students. At this time, students were also provided with a consent form to be filled by the parent/legal guardian. Parents were asked to approve the questionnaire as part of their consent form and to fill the parent questionnaire.

Students were asked to return the consent form and parent questionnaires filled within 3 days. Consent forms were collected by the teachers and stored in a sealed envelope and researchers received the anonymous questionnaires separately. After 3 days, students were invited to participate in the study by returning the written consent form and the parent questionnaires. The student questionnaires were all self-reported and students were instructed to fill them alone without any help from the parents. For 44% of the schools, students were invited to complete the questionnaires in the classroom in the presence of a researcher (researcher-assisted arm) while for the remaining 56% of the schools, students were instructed to fill the questionnaires at home (at-home arm). In both arms, the students filled the questionnaires alone. School allocation to researcher-assisted or home-based data collection was done randomly. The only differentiation between the two study arms is the collection of anthropometry data which were self-reported for the home-based arm and researcher measured in the researcher assisted arm.

The study recruited 1345 participants (45.1% boys), from five regions of Greece (i.e., Attica, Macedonia, Peloponnesus, Thessaly, Thrace, and Crete). The study had a participation rate of 30.1%, based on the number of students who returned a signed consent form. School Lunch recipients (n = 609) were recruited in the May–June from 45 schools and control participants (n = 736) were recruited in the September–October period from 24 schools from the same regions and vicinity. Participants were school-age children enrolled in the fifth and sixth grade (i.e., aged 11 to 12 years old) and their parents. 

#### 2.1.1. Dietary Assessment

Dietary assessment was based on a semi-quantitative food frequency questionnaire (FFQ) which contained 48 food groups commonly used in the local cuisine and was validated for the study age group as a self-completed tool [13]. The FFQ included a picture of an indicative portion size per food group, which was used to quantify the portion size usually consumed by the children (quantification relative to the indicative portion size). A separate set of questions were used to evaluate intake of specific foods, especially whole wheat bread, low fat dairy products, and sugar free soft drinks. Participants were asked to report their usual intake over the past month as ‘daily’, ‘3–6 times per week’, ‘2 times per week’, ‘once a week’, ‘1–2 times per month’, and ‘seldom/never’.

Quantitative analysis of the FFQ into energy, macronutrient, fiber and sodium intake was performed using data from the USDA National Nutrient Database [14] and Hellenic Health Foundation [15]. Diet quality expressed as intakes of key food groups in portion per day was performed against the National Nutrition Guide [16]. 

Over- and under-reporting was assessed using the Goldberg cut-off. [17]. In particular, Basal Metabolic Rate (BMR) was calculated using the Schofield equations and all energy intake to BMR ratios <1.16 and > 2.65 were excluded from the analysis. [18].

Mediterranean diet adherence was evaluated via the KIDMED score (Mediterranean Diet Quality Index for children) [19], which includes 16 dichotomous questions resulting to a score of 0–16. The score is then grouped in classes of ≥8, 4–7, and ≤3, indicative of good, average, and low adherence to the principles of the Mediterranean Diet, respectively.

#### 2.1.2. Anthropometric Data 

For the schools that participated in the researcher-assisted arm, a trained researcher measured anthropometry at the time of the interview (approximately 2 h after the first meal and before the consumption of mid-day snack). Weight was measured with the use of a digital scale (Tanita TBF 300). Students were dressed but removed their shoes. One kilogram was subtracted from all subjects to account for clothing. Standing height was measured using a portable stadiometer (Leicester height-measure) to the nearest 0.1 cm without shoes, with the head positioned according to the Frankfort plane position. In the at-home arm, all anthropometry was self-reported. 

Body mass index (BMI) was calculated by dividing weight (kg) by standing height squared (m^2^). Obesity, overweight and underweight among children were evaluated using sex and age appropriate z-scores on the WHO growth charts [20]. 

#### 2.1.3. Sedentary Lifestyle Evaluation

Sedentary lifestyle was assessed via sleep duration, studying duration, and screen time. All sedentary lifestyle habits were assessed separately for weekdays and weekends. Children were asked to report the hours spent in front of a screen, the hours spent studying for school or other extra-curricular activities. Sleep duration was calculated via questions about their falling asleep and waking up cycle. Children were also asked to report any mid-day naps. Watching television/DVD/movies and/or recreational usage of games consoles/computer was defined as screen time. Children were also asked to report having access to screen (mobile, TV, computer, etc.) in their own bedroom. 

#### 2.1.4. Socio-Economic Status (SES)

An adaptation of the Family Affluence Scale (FAS) [21] was used to define family SES as it has been previously validated and used in Greece [22]. The original FAS evaluates: (i) the number of cars per family, (ii) whether each child in the family has his/her own bedroom, (iii) the number of personal computers their family owns, and (iv) the days spent οn family vacation the past 12 months (to identify higher-SES families in affluent countries). In the current study, parents and children provided data on the first three components of the FAS. However, the Ministry of Education, Department of Primary Education did not allow for the collection of information on family vacation. Hence the adapted FAS score used in this study assumed days spend in vacation to be zero for all participants. As a result, the adapted FAS score in this study is used purely to detect differences among participants and not as an absolute value. 

#### 2.1.5. Parental Data

Information on socio-economic and demographic characteristics, such as parents’ age, current weight and height, years of education, annual family income, employment status, and profession were collected via a questionnaire, which was attached to the consent form. Parents were also asked about the children’s nutrition information (e.g., children preferences on foods, frequency of meals consumed with the family, and the frequency of meals ‘out of home’, who is cooking at home, etc.). Parental dietary habits were assessed using the MedDietScore questionnaire, a measure of adherence to the Mediterranean Diet [22]. Parental obesity and overweight were estimated from self-reported values of body weight and height using standard cut-off values: obesity (BMI ≥ 30.0 kg/m^2^) and overweight (BMI = 25.0–29.9 kg/m^2^) according to the WHO classification [23]. 

### 2.2. Statistical Analysis 

Normal distribution of all continuous variables was tested with P-P test plots and graphically assessed by histograms. Continuous variables are expressed as mean ± standard deviation for variables following normal distribution and median and (Quartile 1, Quartile 3) values if not normally distributed. Differences between groups were tested through independent samples *t*-test and Mann–Whitney U test for normally and non-normally distributed variables accordingly. Linear regression models were used to measure the relationship between dietary habits and sedentary lifestyle parameters. All models were adjusted for residency location, age, sex, socioeconomic level, daily energy intake, and BMI classification. Estimated associations are described in terms of β coefficients and 95% confidence intervals (linear regression models). Collinearity diagnostics were performed using variance inflation factor (VIF) and tolerance values. Significance level was set at 5%. Statistical analysis was performed by SPSS package, version 16.1 (SPSS Inc., Chicago, IL, USA). A sensitivity analysis was performed to check for differences between the two arms of data collection (at-home vs. researcher-assisted).

## 3. Results

Students recruited were primarily female (54.9%) from primarily urban (~75% for both population groups, *p =* 0.924). Children recruited in both groups were 11.45 ± 0.5 years old as per study design without any difference between the two groups (*p =* 0.54, data not shown). No differences were seen in the mean household SES between the two groups (Table 1). In both population groups, mothers were the main responders for the parental questionnaire (~81%). The mean age of mothers was 41.8 ± 5.2 years with slightly younger mothers in the School Lunch group, while no difference was seen in the age of fathers between the two groups with a study mean age of fathers of 45.5 ± 5.8 years (Table 1). Parents in the control group reported higher educational level (in years) compared to the School Lunch group (*p =* 0.02 for both parents).

Although child BMI did not differ between the two groups (*p =* 0.35); a BMI z-score indicative of underweight was more common for children in the School Lunch group, while children in the control group had 8-times higher prevalence of BMI z-score indicative of obesity (Table 1). As far as parental BMI is concerned, no differences were observed in mean BMI of fathers, but mothers in the School Lunch group had higher BMI than the mothers in the Control group (*p =* 0.02). No differences in reported weight, height, and calculated BMI z-score were documented between self-reported (at home collection) and measured values (researcher-assisted collection) (data not shown).

Children in the School Lunch groups consumed fewer meals daily (*p =* 0.02) and were less likely to consume breakfast daily (*p =* 0.03) compared to the control group (Table 2). The latter was mainly due to differences in the frequency of breakfast consumption among boys (*p =* 0.0012) as no differences were seen for girls. Children in the School Lunch group were more likely to have a TV in their own bedroom and spend more hours watching TV or playing video games both during the week and the weekend (*p =* 0.03, ≤0.001, respectively). Gender was an important factor behind those differences as both male and female students in the School Lunch group reported higher weekday screen time compared to the Control group (*p <* 0.001, data not shown) but girls in the Control group reported the lowest screen time during both weekdays and weekends to all other groups. No differences were observed concerning the hours spent studying during the week and weekends, or the frequency of having a habit of watching TV whilst eating meals (Table 2). 

**Figure 1:**
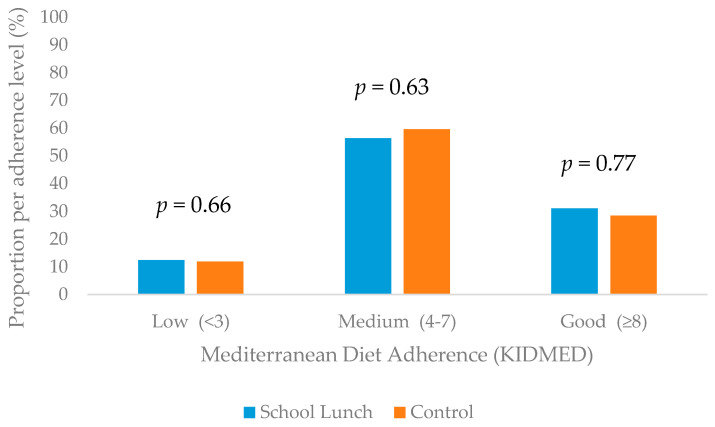
Adherence to Mediterranean Diet of children participants.

The majority of children in both groups showed good adherence to the Mediterranean Diet (School Lunch: 56.4%, Control: 59.6%, *p =* 0.63). Low adherence to the Mediterranean Diet was only reported by 12.5% and 12% of the children in the School Lunch and control group, respectively (Figure 1).

Dietary intake, daily energy, and carbohydrates and protein intake were similar for children in the two groups and both intakes were within the range of respective recommendations (Table 3). Fat consumption and SFA, PUFA, and MUFA intakes were similar for both populations. Children in the School Lunch group consumed higher amounts of sodium (*p =* 0.08) and lower amounts of total sugars (Table 3) irrespective of the total sugar source (sugars from fruits, *p =* 0.08; sugars from dairy, *p =* 0.02; sugars from other sources, *p <* 0.01, data not shown). Sex was a differentiating factor in dietary intake only the case of energy intake (*p <* 0.01), sodium (*p <* 0.01), and calcium (*p <* 0.01), with boys in the control group having different intakes compared to the girl in the same group (Table 3).

At the food group level, children in the School Lunch Program consumed more cereals–potatoes (*p =* 0.02) and legumes (*p =* 0.02), than the Control group but also reported higher intakes of fruits (*p =* 0.03). Both reported average intakes of cereals–potatoes, legumes, vegetables, dairies, fish and seafood, oils-nuts and eggs lower than recommended according to the National Nutrition Guide [16]. Fruit intake was below recommendation only for the control group but not the School Lunch group (Table 4).

A linear regression analysis (Table 5) suggests that participating in the School Lunch Program had no effect on the dietary choices of the children participated in the study. However, the time spent on screens during the week showed a positive association with fast food consumption and the screen time during the weekend was negatively associated with the KIDMED score but positively with the number of portions of fruit consumed (all associations adjusted for sex, BMI, SES, and geographical location of the school). Similarly, sleep duration during the weekends was positively associated with the KIDMED score irrespectively of the screen time during the same days. For every hour of extra sleep during the weekend the intake of legumes reported was 0.23 portions higher and the exact same association was seen for sleep duration during the week and these associations were independent amongst them. Sleep duration during the week was also associated with better nutritional habits in terms of nuts and soft drinks consumption. None of the models reached statistical significance for fish and egg consumption. Interestingly, meat consumption was negatively associated with SES status.

## 4. Discussion

This study is the first to evaluate the dietary and lifestyle habits of children enrolled in the national School Lunch Program in Greece and compare them with an age-socioeconomic status–matched control population of non-recipients. At the same time, this is one of the few epidemiological studies reporting the eating habits of children in Greece after the economic crisis, covering urban and rural environments representative of the population distribution within Greece. 

The most important finding of our study is children enrolled in the School Lunch Program did not report any differences in Mediterranean Diet adherence or the intake of the majority of macronutrients as compared to control subjects. On the other hand, children in the School Lunch group reported lower likelihood of daily breakfast consumption and lower intakes of cereals and legumes, but higher fruit intake, similar to the results reported by adult recipients of the equivalent FEAD program in Greece [12]. However, when adjusted for potential confounders, those differences were shown to be independent on the School Lunch enrollment and more likely to be attributed to differences in sedentary lifestyle parameters (namely screen time, sleep duration) and socioeconomic status. In fact, the most apparent difference between the two groups were the larger duration of time spend in front of a screen both during the week and the weekend for children in the School Lunch group. 

Although large differences were not observed in the dietary habits of the two groups, it is important to highlight that when dietary quality is assessed, both groups showed great room for improvement. For both groups, intakes of energy, carbohydrates, and protein are within the recommended range from EFSA indicating a general food security [24,25,26]. On the other hand, intakes of total fat were higher than the 20–35% of the total daily energy intake according to EFSA, as were SFA [26]. PUFA intakes were lower than the recommended limits and MUFA intakes were within the range of recommendations [26]. Intakes of total sugars were also higher than the 10% of the total daily energy recommended by WHO [27]. On the other hand, sodium intakes were below the 2g/day EFSA recommendation for the specific age-group [28]. Similarly, as shown in Table 4, only meat intake is within the recommended levels and intakes of cereal-potatoes, legumes, vegetables, dairy, seafood and eggs should all be improved. At the level of food groups, the School Lunch group did show larger deviation from the recommendation and hence a lower diet quality, with the exception of fruit intake, in which those in the School Lunch group showed higher adherence to the guidelines. These data are in agreement with previous reports in the same population [29], that indicate a range of ‘hidden sources’ of sodium and SFA especially dairy products (feta cheese) in school-aged children in Greece. Similarly, higher intakes of fruit and vegetables among the lower socioeconomic classes are reported in Greece and Europe attributed to an already high consumption of domestically produced foods [30,31]. Previous analyses of School Lunch Programs showed larger consumptions of meat and fish among the students enrolled in such programs and consumption of larger portions a finding that was not confirmed in our analysis [8], potentially linked to the menu employed in the Greek School Lunch Program which was designed according to the Mediterranean Diet principles.

The second important finding of the study is that children in the School Lunch group were more likely to be classified as underweight compared to the control group. This effect is further enhanced if sex-specific prevalence is calculated, where it becomes apparent that boys are disproportionately affected and especially boys in the School Lunch group (Underweight prevalence in School Lunch group: 26.5% in boys and 4.5% in girls; Control group: 18.1% in boys and 4.6% in girls, Table 1). At the same time, only the 1.3% of children in the School Lunch group and 6.4% in the Control group were classified as overweight/obese, when studies carried prior to the economic crisis reported a very different picture with 4.2% underweight and 41.2% overweight/obese prevalence [32]. This BMI redeployment suggests as 10-fold reduction in overweight/obesity prevalence. Similar BMI redeployments and underweight prevalence of ~9–10% have previously been reported from an epidemiological study conducted in the midst of the economic crisis in 2015 in a similar population [33]. However, due to the nature of the study, such findings will need to be further investigated in nationally representative samples to be confirmed. More specifically, future studies would need to address any potential self-selection bias or the likelihood of miss-reporting of weight and heigh values through direct measurements. In the current study, both self-reported and measured values of weight and height were used and although a sensitivity analysis did not show any significant differences between the two methodologies used, the study could still be susceptible to miss-reporting. 

In terms of lifestyle characteristics, half the population of our study reported sharing at least on meal daily with the family, father or mother (Table 1). Interestingly, the majority of the children (approx. 60%) in both groups also reported a rare occurrence (less than once a week) of having a meal in front of a screen (Table 1). As food consumption in front of a screen is associated with lower diet quality [34] and higher obesity prevalence [35,36,37,38], this indicates the existence of healthy family eating habits in the majority of the households studied, potentially linked to the traditional Mediterranean lifestyle and a slower transition towards more westernized eating habits. Similarly, a slow transition in screen viewing habits was also seen as reported time spent in front of screen has remained unchanged in comparison to data from ~10 years ago [32,38]. 

Another important finding in our study is the substantial increase in proportion of school aged children reporting high adherence to the Mediterranean Diet. Only a small proportion of children in our study (~10%) reported low adherence to the Mediterranean Diet, a significant change to the findings of studies like GRECO and PANACEA reporting large prevalence of low adherence to the Mediterranean Diet prior to the economic crisis [32,39]. This positive transition has been reported previously [40] and—when assessed together with other lifestyle parameters—shows a tendency towards a more traditional lifestyle with the potential to improve health.

Contrary to the positive changes in Mediterranean Diet adherence and screen viewing, around 50% of children in the School Lunch group and more than 40% of children in the Control group reported skipping breakfast frequently a finding unchanged over time [33]. What is more, children in the School Lunch group were still more likely to have less meals during the day (47% reported having 1–3 meals), a finding which suggests a potentially persistent impact of financial necessity towards meal skipping [41].

When studying the results of the current survey in the larger context, our findings are in agreement with previous reports highlighting the capacity of School Lunch Programs to alleviate social disparities in food and nutrition seen in students receiving either a school lunch or breakfast [4,5,6,7]. This study was designed as a cross-sectional comparison between children who were enrolled in the School Lunch Program versus children in the same vicinity that did not receive such an intervention. Operating under the hypothesis that the School Lunch Program is available to children at an increased risk of undernutrition, based on unemployment and deprivation data, the study aimed to provide an indication of the capacity of the School Lunch Program to address said risk. Our findings indicate clear differences in lifestyle parameters such as screen time between the two groups but less clear differences in the nutritional habits. The lack of such differences could be interpreted as a success for the program to remove the risk of undernutrition but it could also be due to no prior differences between the two groups, in which case the program has limited to no impact. Unfortunately, the lack of longitudinal data does not allow for the measurement of the direct impact on the School Lunch Program on dietary intakes of its recipients. However, it could be stipulated that the School Lunch Program is at least partially contributing to this lack of difference in the dietary intake of children. Previous results from a similar analysis in adults receiving state food-bank aid showed that differences in dietary intakes persisted despite being enrolled in the program, which was attributed to the low contribution of the food bank program to the daily energy intake [12].

This study is not free of limitations. The combination of researcher assisted on site and self-reported data collection at home has the capacity to introduce errors. The major concern for these types of errors were deliberate or involuntary mistakes in dietary exposure assessment. To remedy such errors, the Goldberg cut-offs were employed to identify and exclude extreme values. In fact, there was a larger proportion of extreme values in the at-home module as opposed to the researcher-assisted module mainly due to poor understanding of the FFQ completion (issues with serving size declaration, data not shown). The second limitation is linked with the choice of dietary assessment methods. The choice of a semi-quantitative FFQ, although suitable for the purpose of the study, does not allow for a detailed analysis of the specific foods consumed. Despite potential errors in their accuracy, the use of self-reported anthropometric data and self-reported dietary intake data through a semi quantitative FFQ should not be considered as a substantial source of bias in this analysis, as the main objective was to identify differences between two population groups assessed under the same conditions and using the same tools.

## 5. Conclusions

This is the first in-depth analysis of the dietary and lifestyle characteristics of school-aged children in the post-economic crisis Greece. Overall, children reported low adherence to the National Dietary Guidelines, but dietary choices which were in line with the Mediterranean Diet. Children receiving a school lunch show little to no difference in dietary habits to their age and vicinity matched counterparts. Differences in lifestyle habits however persist. In fact, sleep duration and screen time were highlighted as the main contributors towards differences in dietary habits.

## Figures and Tables

**Table 1:** Sociodemographic and anthropometric characteristics of children and their parents per population group.

Characteristics	School Lunch Group	Control Group	*p*
Children (*n*)	595	736	
Weight (kg)	43.86 ± 10.82	41.55 ± 9.57	<0.001
Height (cm)	149.47 ± 8.97	147.33 ± 8.44	<0.001
BMI (kg/m^2^)	19.18 ± 3.44	19.37 ± 3.68	0.35
Underweight % (n)	14.6 (78)	10.7 (73)	<0.001
Normal weight % (n)	84.1 (451)	82.8 (565)	
Overweight % (n)	0.6 (3)	0.7 (5)	
Obese % (n)	0.7 (4)	5.7 (39)	
SES (2–16)	6.45 ± 1.62	6.35 ± 1.89	0.52
Parents (*n*)	203	305	
Mothers *n* (%)	165 (80.8)	247 (81.0)	
Mothers’ age (years)	41.02 ± 5.46	42.26 ± 5.05	0.01
Fathers; age (years)	46.49 ± 5.77	45.57 ± 5.84	0.88
Mothers’ education (years of school)	13.30 ± 3.09	14.09 ± 3.77	0.02
Fathers’ education (years of school)	12.64 ± 3.18	13.45 ± 3.90	0.02
BMI of mothers (kg/m^2^)	25.08 ± 4.50	24.14 ± 4.17	0.02
BMI of fathers (kg/m^2^)	25.52 ± 3.41	27.23 ± 5.27	0.52

*p* refers to comparisons between School Lunch and Control group; *p*-values are derived via the independent samples *t*-test.

**Table 2:** Eating habits and sedentary lifestyle characteristics of the population.

	School Lunch Group			Control Group			*p*
	Boys (*n* = 269)	Girls (*n* = 326)	Total (*n* = 595)	Boys (*n* = 331)	Girls (*n* = 405)	Total (*n* = 736)	School Lunch vs. Control
Number of eating occasions (times/day)	3.41 ± 1.36	3.52 ± 1.41	3.47 ± 1.38	3.67 ± 1.36	3.64 ± 1.33	3.65 ± 1.35	0.02
Having breakfast (times per week)	4.90 ± 2.37	5.04 ± 2.36	4.98 ± 2.37	5.28 ± 2.27	5.17 ± 2.31	5.22 ± 2.29	0.06
Eating breakfast daily (%)	49.0	53.0	51.2	58.8	55.1	56.7	0.026
Having meal with family, father or mother (%)							
Rarely	4.7	8.2	6.6	5.5	5.3	5.4	0.22
1–2 times/week	16.7	16.3	16.5	22.1	22.1	22.1	
3–4 times/week	15.6	12.2	13.7	16.3	14.0	15.0	
5–6 times/week	17.9	12.5	14.9	8.6	11.0	9.9	
Daily	45.1	50.8	48.3	47.2	47.6	47.4	
Having TV in the bedroom (%)	41.6	33.4 *	37.1	36.8	27.0 *	31.4	0.03
Having PC/video game player in the bedroom (%)	61.4	55.4	58.1	51.1	58.5 *	55.2	0.30
Studying hours (weekdays)	2.07 ± 1.11	2.25 ± 1.16 *	2.17 ± 1.14	2.13 ± 1.02	2.27 ± 1.12	2.21 ± 1.08	0.57
Studying hours (weekends)	2.19 ± 2.11	2.22 ± 2.31	2.21 ± 2.22	2.22 ± 2.26	2.13 ± 1.54	2.17 ± 1.90	0.74
TV/video game weekdays (h)	2.32 ± 2.74	2.10 ± 2.47	2.20 ± 2.59	1.58 ± 1.30	1.46 ± 0.99	1.52 ± 1.14	<0.001
TV/video game weekends (h)	3.87 ± 2.83	3.50 ± 2.86	3.66 ± 2.85	3.59 ± 2.49	2.85 ± 1.77 *	3.19 ± 2.16	0.001
Having meal while watching TV or playing video games (times) (%)							
Rarely	30.9	31.5	31.2	37.5	33.8	35.5	0.99
1–2 times/week	34.0	32.8	33.3	26.8	31.6	29.4	
3–4 times/week	16.0	14.8	15.4	13.2	12.2	12.7	
5–6 times/week	5.5	6.3	5.9	5.8	7.1	6.5	
Daily	13.7	14.5	14.1	16.3	15.3	15.7	

Results are presented as the mean ± SD for the normally distributed variables and as P50 (P25, P75) for non-normally distributed variables. *p* refers to comparisons between total School Lunch and Control group, * *p* < 0.05 indicates significant difference between boys and girls within each group. *p*-values are derived via the independent samples *t*-test or Mann–Whitney U for continuous variables and chi-square for nominal variables.

**Table 3:** Daily energy, macronutrients, sodium, calcium, fibers, and sugars intake of children participants, according to population group and gender.

	School Lunch Group			Control Group			
	Boys (*n* = 101)	Girls (*n* = 97)	Total (*n* = 197)	Boys (*n* = 129)	Girls (*n* = 156)	Total (*n* = 285)	*p*
Total Energy (kcal)	2163(1549,3746)	2028(1490,2785)	2060(1511,3438)	2236(1597,3170)	1873(1340,2494) *	1977(1498,2700)	0.13
Total carbs (% en)	45.3(39.9,51.64)	46.1 ± 9.1	45.3(39.9,51.6)	44.9(39.3,0.6)	46.0 ± 8.8	45.6 ± 8.5	0.77
Total protein (% en)	15.3(13.6,16.8)	14.7 ± 2.9	15.1 ± 3.1	15.4(13.7,17.3)	15.0 ± 3.1	15.2 ± 2.9	0.72
Total fat (% en)	41.8 ± 7.9	41.9 ± 7.3	41.8 ± 7.6	41.4(38.5,46.3)	41.9(37.9,45.3)	41.7 ± 6.1	0.84
SFA (% en)	14.4(12.3,17.2)	14.3(12.5,16.3)	14.7 ± 3.7	14.8(12.3,16.7)	13.89(12.04,16.46)	14.5 ± 3.5	0.64
PUFA (% en)	4.92 (4.2,5.6)	5.1(4.2,5.9)	5.0(4.2,5.6)	4.9 ± 1.1	5.2 ± 1.4	5.1 ± 1.3	0.80
MUFA (% en)	18.4 ± 5.1	17.8(15.6,20.9)	18.6 ± 5.1	17.62(15.9,20.6)	18.7(15.9,21.6)	18.0(15.9,21.0)	0.82
Total sodium (mg)	1899(1268,3212)	1726(1193,2412)	1762(1258,2831)	1949(1288,2840)	1533(997,2224) *	1618(1153,2443)	0.08
Total calcium (mg)	1392(888,2096)	1234(854,1674)	1303(871,1889)	1215(875,2183)	1063(691,1611) *	1150(809,1819)	0.13
Total fiber (g/d)	4.5 ± 1.4	4.7 ± 1.2	4.6 ± 1.3	4.3(3.6,5.2)	4.6 ± 1.2	4.4(3.7,5.3)	0.61
Total sugars (% en)	22.4(19.0,28.6)	23.3(18.9,29.5)	22.8(18.9,28.9)	21.8(18.3,25.7)	23.0(18.4,27.1)	22.9 ± 7.2	0.22

Results are presented as the mean ± SD for the normally distributed variables and as P50 (P25, P75) for non-normally distributed variables. *p* refers to comparisons between total School Lunch and Control group, * *p* < 0.05 indicates significant difference between boys and girls. *p*-values are derived via the independent samples *t*-test for the normally distributed variables and via the Mann–Whitney U-test for skewed variables. % en: values are expressed as % of the daily energy intake.

**Table 4:** Daily intake from food groups in the School Lunch and the Control group and in comparison with the National Nutrition Guide.

	National Nutrition Guide	School Lunch Group (Portions/Day)	Control Group (Portions/Day)	*p*
Cereals-Potatoes (rice, pasta, breakfast cereals, bread, pastries, toast, crackers, potatoes)	5–6 portions/day	1.40(0.93,2.36)	2.12 ± 1.40	0.02
Legumes (lentils, beans, chickpeas)	at least 3 portions/week (0.43 portions /day)	0.27(0.13,0.53)	0.33(0.13,0.53)	0.02
Fruits (raw, dried, fresh juices)	2–3 portions /day	2.29 ± 1.59	1.66(0.90,2.53)	0.03
Vegetables (raw and cooked)	2–3 portions /day	0.67(0.40,1.10)	0.83(0.40,1.37)	0.15
Dairy (milk, yogurt, cheese, dairy products)	3–4 portions /day	1.03(0.50.1.74)	1.19(0.73,1.73)	0.18
Meat (beef, beef, chicken, turkey)	2–3 portions/week (0.36 portions /day)	0.40(0.27,0.66)	0.40(0.27,0.80)	0.32
Fish and Seafood	2–3 portions/week (0.36 portions /day)	0.13(0.03,0.27)	0.13(0.03,0.27)	0.85
Fast foods	-	0.49(0.30,0.90)	0.53(0.26,0.96)	0.91
Oils and Nuts (nuts, olive oil, butter, margarine)	3–4 portions /day	2.67 ± 1.56	2.55 ± 1.40	0.46
Eggs	4–7 portions/week (0.78 portions /day)	0.27(0.07,0.53)	0.27(0.07,0.53)	0.43
Soft drinks	-	0.03(0.03,0.13)	0.03(0.00,0.13)	0.05
Sweets (sugar, honey, jam, chocolate)	-	1.26(0.80,1.93)	1.180(0.70,2.00)	0.62

Results are presented as the mean ± SD for the normally distributed variables and as P50 (P25, P75) for non-normally distributed variables. *p* refers to comparisons between School Lunch and Control group, *p*-values are derived via the independent samples *t*-test for the normally distributed variables and via the Mann–Whitney U-test for skewed variables.

**Table 5:** Linear regression models to evaluate sociodemographic, anthropometric, and dietary habits factors with KIDMED and food categories consumption.

	KIDMED	Fruits	Vegetables	Legumes	Fast Food	Meat	Cereals	Nuts	Soft Drinks	Sweets
	β (95% CI)	β (95% CI)	β (95% CI)	β (95% CI)	β (95% CI)	β (95% CI)	β (95% CI)	β (95% CI)	β (95% CI)	β (95% CI)
School Lunch enrollment	0.042 (−0.463 to 0.864)	0.027 (−0.496 to 0.718)	−0.06 (−0.276 to 0.254)	−0.016 (−0.168 to 0.135)	−0.122 (−0.616 to 0.038)	−0.052 (−0.242 to 0.111)	−0.110 (−0.759 to 0.879)	0.60 (−0.298 to 0.879)	0.111 (−0.015 to 0.129)	0.046 (−0.886 to 1.788)
Periphery	0.004 (−0.105 to 0.111)	−0.038 (−0.120 to 0.072)	0.029 (−0.034 to 0.050)	−0.046 (−0.031 to 0.017)	−0.016 (−0.058 to 0.046)	0.009 (−0.026 to 0.030)	0.076 (−0.032 to 0.103)	0.056 (−0.052 to 0.134)	−0.080 (−0.018 to 0.005)	−0.031 (−0.258 to 0.165)
Sex	0.123 (−0.116 to 1.275)	0.018 (−0.553 to 0.699)	0.041 (−0.201 to 0.346)	−0.048 (−0.206 to 0.108)	−0.025 (−0.397 to 0.278)	−0.024 (−0.212 to 0.151)	0.108 (−0.117 to 0.764)	0.154 (0.135 to 1.348) *	−0.089 (−0.120 to 0.029)	0.103 (−0.375 to 2.382)
SES	−0.049 (−0.254 to 0.128)	0.041 (−0.129 to 0.223)	−0.006 (−0.080 to 0.074)	−0.002 (−0.045 to 0.043)	0.059 (−0.056 to 0.134)	−0.159 (−0.107 to −0.005) *	0.050 (−0.082 to 0.165)	0.025 (−0.137 to 0.204)	0.046 (−0.014 to 0.028)	−0.074 (−0.587 to 0.188)
Screen time weekdays	−0.194 (−0.641 to 0.025)	0.067 (−0.209 to 0.399)	−0.045 (−0.161 to 0.105)	0.072 (−0.050 to 0.102)	0.238 (0.032 to 0.360) *	−0.037 (−0.104 to 0.072)	−0.044 (−0.260 to 0.168)	−0.028 (−0.341 to 0.248)	0.031 (−0.031 to 0.042)	0.024 (−0.588 to 0.751)
Screen time weekends	−0.268 (−0.445 to −0.064) *	0.034 (−0.147 to 0.205) *	−0.071 (−0.103 to 0.051) *	−0.027 (−0.050 to 0.038)	−0.102 (−0.145 to 0.044)	0.162 (−0.008 to 0.094)	0.055 (−0.089 to 0.158)	0.029 (−0.141 to 0.199)	−0.017 (−0.023 to 0.019)	−0.064 (−0.516 to 0.257)
Sleep duration weekdays	−0.102 (−0.623 to 0.117)	0.116 (−0.084 to 0.600)	0.065 (−0.086 to 0.212)	0.237 (0.047 to 0.218) *	−0.009 (−0.196 to 0.172)	−0.018 (−0.111 to 0.087)	0.134 (−0.020 to 0.460)	0.164 (0.097 to 0.759) *	−0.177 (−0.090 to −0.009) *	−0.033 (−0.925 to 0.579)
Sleep duration weekends	0.165 (0.014 to 0.180) *	−0.056 (−0.106 to 0.047)	0.003 (−0.033 to 0.034)	0.227 (0.011 to 0.049) *	−0.018 (−0.047 to 0.036)	0.029 (−0.018 to 0.027)	−0.035 (−0.067 to 0.040)	0.026 (−0.058 to 0.090)	0.115 (−0.002 to 0.017)	−0.026 (−0.202 to 0.136)
Energy intake	0.159 (0.00 to 0.001) *	0.258 (0.00 to 0.001) *	0.246 (0.00 to 0.00) *	0.010 (0.00 to 0.00)	0.004 (0.00 to 0.00) *	0.375 (0.00 to 0.00) *	0.369 (0.00 to 0.001) *	0.592 (0.001 to 0.001) *	0.307 (0.00 to 0.00) *	0.459 (0.001 to 0.002) *
BMI category	0.029 (−0.267 to 0.395)	−0.036 (−0.366 to 0.224)	0,158 (0.005 to 0.262) *	0.048 (−0.050 to 0.097)	−0.026 (−0.118 to 0.129)	−0.027 (−0.102 to 0.069)	0.015 (−0.186 to 0.229)	0.050 (−0.172 to 0.399)	−0.040 (−0.045 to 0.025)	0.006 (−0.622 to 0.676)

* *p* < 0.05 indicates significant difference; CI, Confidence interval.

## Data Availability

The data presented in this study are available on request from the corresponding author. The data are not publicly available due to the confidential nature of some information.

## References

[1] Lucas P.J., Patterson E., Sacks G., Billich N., Evans C.E.L. (2017). Preschool and School Meal Policies: An Overview of What We Know about Regulation, Implementation, and Impact on Diet in the UK, Sweden, and Australia. Nutrients.

[2] Oostindjer M.A.-O., Aschemann-Witzel J., Wang Q., Skuland S.E., Egelandsdal B., Amdam G.V., Schjøll A., Pachucki M.C., Rozin P., Stein J. (2017). Are school meals a viable and sustainable tool to improve the healthiness and sustainability of children’s diet and food consumption? A cross-national comparative perspective. Crit. Rev. Food Sci. Nutr..

[3] McLoughlin G.M., Turner L., Leider J., Piekarz-Porter E., Chriqui J.F. (2020). Assessing the Relationship between District and State Policies and School Nutrition Promotion-Related Practices in the United States. Nutrients.

[4] Vernarelli J.A., O’Brien B. (2017). A Vote for School Lunches: School Lunches Provide Superior Nutrient Quality than Lunches Obtained from Other Sources in a Nationally Representative Sample of US Children. Nutrients.

[5] Vik F.N., Van Lippevelde W., Øverby N.C. (2019). Free school meals as an approach to reduce health inequalities among 10–12- year-old Norwegian children. BMC Public Health.

[6] Μoore G.M., Murphy S., Chaplin K., Lyons R.A., Atkinson M., Moore L. (2019). Selection and consumption of lunches by National School Lunch Program participants. Appetite.

[7] Moore G.F., Murphy S., Chaplin K., Lyons R.A., Atkinson M., Moore L. (2014). Impacts of the Primary School Free Breakfast Initiative on socio-economic inequalities in breakfast consumption among 9–11-year-old schoolchildren in Wales. Public Health Nutr..

[8] Huang Z., Gao R., Bawuerjiang N., Zhang Y., Huang X., Cai M. (2017). Food and Nutrients Intake in the School Lunch Program among School Children in Shanghai, China. Nutrients.

[9] Kentikelenis A., Karanikolos M., Papanicolas I., Basu S., McKee M., Stuckler D. (2011). Health Effects of Financial Crisis: Omens of a Greek Tragedy. Lancet.

[10] Anagnostopoulos D.C., Soumaki E. (2013). The state of child and adolescent psychiatry in Greece during the international financial crisis: A brief report. Eur. Child Adolesc. Psychiatry.

[11] Alderman L. (2013). More Children in Greece Are Going Hungry. The New York Times.

[12] Chatzivagia E., Pepa A., Vlassopoulos A., Malisova O., Filippou K., Kapsokefalou M. (2019). Nutrition Transition in the Post-Economic Crisis of Greece: Assessing the Nutritional Gap of Food-Insecure Individuals. A Cross-Sectional Study. Nutrients.

[13] Farajian P., Karasouli K., Risvas G., Panagiotakos D., Zampelas A. (2009). Repeatability and Validity of a Food Frequency and Dietary Habits Questionnaire in Children. Circulation.

[14] U.S. Department of Agriculture Agricultural Research Service. USDA National Nutrient Database for Standard Reference. https://data.nal.usda.gov/dataset/usda-national-nutrient-database-standard-reference-legacy-release.

[15] Hellenic Health Foundation Nutritional Composition Tables οf Greek Recipes by Calculation. http://www.hhf-greece.gr/tables/DishesIntro.aspx?l=en.

[16] Yannakoulia M.L.A., Kastorini C.M., Saranti Papasaranti E., Petralias A., Veloudaki A., Linos A. (2014). DIATROFI Program Research Team. National Nutrition Guide. http://www.diatrofikoiodigoi.gr/?Page=english-menu.

[17] Black A.E. (2000). Critical evaluation of energy intake using the Goldberg cut-off for energy intake:basal metabolic rate. A practical guide to its calculation, use and limitations. Int. J. Obes..

[18] Schofield W.N. (1985). Predicting basal metabolic rate, new standards and review of previous work. Hum. Nutr Clin. Nutr..

[19] Serra-Majem L., Ribas L., Ngo J., Ortega R.M., García A., Pérez-Rodrigo C., Aranceta J. (2007). Food, youth and the Mediterranean diet in Spain. Development of KIDMED, Mediterranean Diet Quality Index in children and adolescents. Public Health Nutr..

[20] World Health Organization BMI-for-Age (5–19 Years). https://www.who.int/tools/growth-reference-data-for-5to19-years/indicators/bmi-for-age.

[21] Currie C., Molcho M., Boyce W., Holstein B., Torsheim T., Richter M. (2008). Researching health inequalities in adolescents: The development of the Health Behaviour in School-Aged Children (HBSC) family affluence scale. Soc. Sci. Med..

[22] Panagiotakos D.B., Pitsavos C., Stefanadis C. (2006). Dietary patterns: A Mediterranean diet score and its relation to clinical and biological markers of cardiovascular disease risk. Nutr. Metab. Cardiovasc. Dis..

[23] World Health Organization (1997). Obesity: Preventing and Managing the Global Epidemic. Report of a WHO Consultation on Obesity.

[24] Arsenault J.E., Brown K.H. (2017). Dietary Protein Intake in Young Children in Selected Low-Income Countries Is Generally Adequate in Relation to Estimated Requirements for Healthy Children, Except When Complementary Food Intake Is Low. J. Nutr..

[25] Graham G.G., MacLean W.C., Brown K.H., Morales E., Lembcke J., Gastañaduy A. (1996). Protein requirements of infants and children: Growth during recovery from malnutrition. Pediatrics.

[26] EFSA Dietary Reference Values (DRVs). https://www.efsa.europa.eu/en/interactive-pages/drvs.

[27] World Health Organization, World Health Organization (2015). Guideline: Sugars Intake for Adults and Children.

[28] Castenmiller J., de Henauw S., Hirsch-Ernst K.-I., Kearney J., Knutsen H.K., Maciuk A., Mangelsdorf I., McArdle H.J., Pelaez C., EFSA Panel on Nutrition, Novel Foods and Food Allergens (NDA) (2019). Dietary reference values for sodium. EFSA J..

[29] Magriplis E., Farajian P., Pounis G.D., Risvas G., Panagiotakos D.B., Zampelas A. (2011). High sodium intake of children through ‘hidden’ food sources and its association with the Mediterranean diet: The GRECO study. J. Hypertens.

[30] Roos G., Johansson L., Kasmel A., Klumbiene J., Prättälä R. (2001). Disparities in vegetable and fruit consumption: European cases from the north to the south. Public Health Nutr..

[31] Trichopoulou A., Naska A., Costacou T., DAFNE III Group (2002). Disparities in food habits across Europe. Proc. Nutr. Soc..

[32] Farajian P., Risvas G., Karasouli K., Pounis G., Kastorini C.-M., Panagiotakos D., Zampelas A. (2011). Very high childhood obesity prevalence and low adherence rates to the Mediterranean diet in Greek children: The GRECO Study. Atherosclerosis.

[33] Yannakoulia M., Lykou A., Kastorini C.M., Papasaranti E.S., Petralias A., Veloudaki A., Linos A. (2016). Socio-economic and lifestyle parameters associated with diet quality of children and adolescents using classification and regression tree analysis: The DIATROFI study. Public Health Nutr..

[34] Trofholz A.C., Tate A.D., Miner M.H., Berge J.M. (2017). Associations between TV viewing at family meals and the emotional atmosphere of the meal, meal healthfulness, child dietary intake, and child weight status. Appetite.

[35] Jones B.L. (2018). Making time for family meals: Parental influences, home eating environments, barriers and protective factors. Physiol. Behav..

[36] Jackson J.A., Smit E., Branscum A., Gunter K., Harvey M., Manore M.M., John D. (2017). The Family Home Environment, Food Insecurity, and Body Mass Index in Rural Children. Health Educ. Behav..

[37] Vik F.N., Bjørnarå H.B., Øverby N.C., Lien N., Androutsos O., Maes L., Jan N., Kovacs E., Moreno L.A., Dössegger A. (2013). Associations between eating meals, watching TV while eating meals and weight status among children, ages 10-12 years in eight European countries: The ENERGY cross-sectional study. Int. J. Behav. Nutr. Phys. Act..

[38] Farajian P., Panagiotakos D.B., Risvas G., Malisova O., Zampelas A. (2014). Hierarchical analysis of dietary, lifestyle and family environment risk factors for childhood obesity: The GRECO study. Eur. J. Clin. Nutr..

[39] Arvaniti F., Priftis K.N., Papadimitriou A., Papadopoulos M., Roma E., Kapsokefalou M., Anthracopoulos M.B., Panagiotakos D.B. (2011). Adherence to the Mediterranean type of diet is associated with lower prevalence of asthma symptoms, among 10-12 years old children: The PANACEA study. Pediatr. Allergy Immunol..

[40] Papadaki S., Mavrikaki E. (2015). Greek adolescents and the Mediterranean diet: Factors affecting quality and adherence. Nutrition.

[41] Sustain 1 in 4 UK Parents Skipping Meals due to Lack of Money. https://www.sustainweb.org/news/jan18_calls_grow_for_government_food_insecurity_measurement/.

